# Opportunities and limits of open partial horizontal laryngectomies for naïve T3–T4a laryngeal cancer: a systematic review and meta-analysis

**DOI:** 10.3389/fonc.2025.1550079

**Published:** 2025-04-28

**Authors:** Erika Crosetti, Andrea Lorenzi, Carmine Prizio, Andrea Elio Sprio, Marco Fantini, Alice Azizi Semeskandi, Andy Bertolin, Giulia Arrigoni, Giovanni Succo

**Affiliations:** ^1^ ENT University Clinic – Head and Neck Cancer Unit, San Giovanni Bosco Hospital, Turin, Italy; ^2^ Department of Surgical Science, University of Turin, Turin, Italy; ^3^ Department of Research, ASOMI College of Sciences, Marsa, Malta; ^4^ Otorhinolaryngology Unit, Vittorio Veneto Hospital, Azienda Unità Locale Socio Sanitaria n. 2. (AULSS2) Treviso, Vittorio Veneto, Italy; ^5^ Department of Oncology, University of Turin, Turin, Italy

**Keywords:** laryngeal cancer, LSCC, partial laryngectomy, open partial horizontal laryngectomy, T3 laryngeal cancer, T4 laryngeal cancer, laryngeal preservation

## Abstract

**Background:**

The present systematic review aims to investigate the survival rates and surgical outcomes of patients with treatment‐naïve, intermediate (T3) to early advanced (T4a) laryngeal squamous cell carcinoma (LSCC) managed with open partial horizontal laryngectomies (OPHLs).

**Methods:**

A systematic literature search was conducted in PubMed, Embase, and Scopus for studies published between January 2000 and December 2023. The Preferred Reporting Items for Systematic Reviews and Meta-Analyses (PRISMA) guidelines were followed. Inclusion criteria were: patients with histopathological confirmed LSCC; tumor classified as T3 or T4a stage according to the American Joint Committee on Cancer (AJCC) staging system; having undergone OPHL as the primary treatment without any prior therapy; availability of at least one of the following outcomes: overall survival (OS), disease-specific survival (DSS), disease-free survival (DFS), local control (LC), locoregional control (LRC), laryngectomy-free survival (LFS), and laryngo-esophageal dysfunction-free survival (LEDFS).

**Results:**

A total of 16 studies were deemed eligible for the qualitative analysis. The cumulative number of patients was 1473. The sample size ranged from 17 to 390 patients. The follow-up period ranged from 0 to 198 months. In patients treated with OPHL for T3, the overall five-year pooled proportions were OS 0.82, DSS 0.88, DFS 0.80, and LFS 0.86, whereas for the T4a case series, they were OS 0.77, DSS 0.89, DFS 0.74, and LFS 0.78.

**Conclusions:**

OPHL for selected T3 and low extralaryngeal volume T4a LSCC can guarantee a high rate of oncological success. Accurate patient selection is paramount to differentiate advanced diseases that is amenable to conservative surgery.

## 1 Introduction

The management of laryngeal squamous cell carcinoma (LSCC) has evolved significantly since the first total laryngectomy was performed in 1873, reflecting a broader trend in oncologic surgery toward function preservation and improved quality of life (1). Historically, total laryngectomy represented the cornerstone of treatment for advanced LSCC, particularly in the T3 and T4a stages. Although effective in disease control, it is associated with significant morbidity—most notably, the requirement for a permanent tracheostomy—which significantly impairs patients’ quality of life.

In recent decades, a shift in treatment paradigms has occurred, particularly for early stage (T1N0 and T2N0) LSCCs, where less invasive procedures and organ-preserving strategies have become the standard of care. These approaches have reduced reliance on total laryngectomy, significantly improving functional outcomes while maintaining excellent oncologic outcomes (2). However, in the context of intermediate and locally advanced LSCCs—particularly in T3 and T4a cases with N+ status—the role of total laryngectomy remains robust. This is particularly true in cases where the tumor exhibits aggressive behavior, locoregional spread, or involvement of critical anatomical structures, where the chances of achieving locoregional control with non-radical treatments are reduced.

Nonetheless, recent advances in surgical techniques and better patient selection have expanded the indications for function-preserving surgeries even in more advanced cases (3, 4). Open partial horizontal laryngectomies (OPHLs) have emerged as a promising alternative. OPHLs allow for the preservation of laryngeal functions, including voice and swallowing, and eliminate the need for a permanent tracheostomy, thus offering a better quality of life post-surgery (5–8). The evolution of OPHL has been marked by a growing body of evidence demonstrating its effectiveness in carefully selected patients with T3N0 and T4aN0 LSCCs, particularly those with limited regional disease and favorable anatomical conditions.

The success of OPHL in managing advanced LSCCs depends on careful patient selection. Studies have shown that in well-selected patients, OPHL can achieve oncologic outcomes comparable to those of total laryngectomy, with five-year overall survival (OS) rates often exceeding 70%, and disease-specific survival (DSS), disease-free survival (DFS), and laryngectomy-free survival (LFS) showing similarly promising results (9, 10).

Considering these developments, this systematic review aims to consolidate the current evidence regarding the outcomes of OPHL in treatment-naïve patients with LSCCs classified as either T3 or T4a, with a low N-stage (N0-1). The review will also critically evaluate the five-year oncologic outcomes, including OS, DFS, DSS, and LFS, focusing on assessing the shift toward function-preserving surgical strategies as an alternative to total laryngectomy in appropriately selected cases. This strategic shift underscores the increasing focus on balancing oncologic control with laryngeal function preservation, ultimately enhancing patients’ overall quality of life with advanced LSCC. This review offers a comprehensive overview of partial laryngeal surgery, shedding light on the evolving landscape of LSCC treatment and establishing OPHL as a conservative yet effective alternative to total laryngectomy.

## 2 Materials and methods

The study strictly adhered to the Preferred Reporting Items for Systematic Reviews and Meta-Analyses (PRISMA) guidelines (11). Ethical approval and informed consent were not required, as all data were derived from previously published literature.

The study selection criteria were defined using the PICOS framework as follows (12, 13):

- Patients (P): adults diagnosed with intermediate (T3) and locally advanced (T4a) LSCC, predominantly with N0 status
- Intervention (I): OPHLs
- Comparator (C): not applicable
- Outcomes (O): overall survival (OS) (primary outcome), disease-specific survival (DSS), disease-free survival (DFS), local control (LC), locoregional control (LRC), laryngectomy-free survival (LFS), and laryngo-esophageal dysfunction-free survival (LEDFS) (secondary outcomes)
- Study design (S): both retrospective and prospective cohort studies were considered

A comprehensive literature search for articles published between 2000 and 2023 was performed on July 31, 2024 using PubMed, Embase, and Scopus. The search strategy combined the following terms: “(larynx OR laryngeal OR glottis OR glottic OR supraglottic OR subglottic) AND (cancer OR carcinoma OR tumor OR neoplasm) AND (T3 OR T4 OR T4a OR pT3 OR pT4 OR pT4a OR intermediate OR advanced) AND (“partial laryngectomy” OR “supracricoid laryngectomy” OR “supraglottic laryngectomy” OR “supratracheal laryngectomy” OR OPHL) NOT (TLM OR TOLM OR transoral OR TORS OR laser OR robotic OR thyroid).” The full text of relevant articles was then screened for inclusion. Additionally, the references of all selected studies were reviewed to identify any further eligible publications. In cases where multiple reports from the same research group or center described potentially overlapping case series, priority was given to the most recent eligible publication.

### 2.1 Eligibility criteria

Three authors (AL, CP, and AAS) independently reviewed all studies identified through the initial literature search. The analysis included an article only if all three reviewers reached a consensus. In cases of uncertainty, the full text was thoroughly examined, and any remaining disagreements were resolved by consultation with two senior authors (GS, EC).

Following PICOS framework, our study focused on patients with LSCC who underwent tumor excision via OPHL as the primary treatment, with no prior therapies (treatment-naïve).

The inclusion criteria were: (*a*) a histopathologically confirmed diagnosis of LSCC, (*b*) tumor staging of T3 or T4a according to the American Joint Committee on Cancer (AJCC) staging system (14), (*c*) OPHL performed as the primary treatment without prior therapy, and (*d*) availability of at least one of the following outcomes: overall survival (OS), disease-specific survival (DSS), disease-free survival (DFS), local control (LC), locoregional control (LRC), laryngectomy-free survival (LFS), and laryngo-esophageal dysfunction-free survival (LEDFS).

The exclusion criteria were: (*a*) duplicate publications, (*b*) studies unavailable in full-text form, (*c*) studies with insufficient or non-extractable data, (*d*) studies focusing on different head and neck malignancies other than LSCC, (*e*) squamous cell carcinoma (SCC) originating from sites other than the larynx, (*f*) patients who received prior therapies before surgery (e.g., induction chemotherapy, radiation therapy, laser surgery), and (*g*) article types including reviews, case reports, conference abstracts, letters to the editor, or book chapters.

Overall survival (OS) was measured from the surgery date to the date of death or the last follow-up.

Disease-specific survival (DSS) was calculated from the date of surgery to the date of cancer-related death or the last follow-up.

Disease-free survival (DFS) was defined as the interval between surgery and the occurrence of local, nodal, or distant recurrence or the last follow-up.

Local control (LC) refers to the time from surgery to the detection of local recurrence or the last follow-up.

Locoregional control (LRC) was defined as the time from surgery to the occurrence of either local or regional recurrence or the last follow-up.

Laryngectomy-free survival (LFS) was measured as the time to laryngectomy, irrespective of the cause (whether functional, due to recurrence, or upfront within the context of primary surgical treatment), or the last follow-up.

Laryngo-esophageal dysfunction-free survival (LEDFS) was defined as the time until any of the following events occurred: death, local recurrence, total laryngectomy, tracheostomy (after more than two years), or gastrostomy tube placement (after more than two years), or until the last follow-up.

### 2.2 Data extraction

The extracted data comprised the following variables: first author, year of publication, patient recruitment method, nationality, number of patients, age, sex, margin status, all available endpoints (OS, DSS, DFS, LC, LRC, LFS, and LEDFS), follow-up duration, neck treatment, adjuvant therapy, TNM stage, and level of evidence (LoE).

### 2.3 Assessment of study quality

Upon completion of data collection, the risk of bias for each study was independently assessed by the Reporting Recommendations for Tumor Marker Prognostic Studies (REMARK) guidelines (15). The REMARK guidelines encompass eight distinct domains, each evaluated as either adequate (scored as 1) or inadequate (scored as 0):

1. Clearly defined inclusion and exclusion criteria
2. Study design (prospective or retrospective)
3. Description of patient characteristics
4. Description of tumor characteristics
5. Definition of margin status
6. Description of study endpoints or outcomes
7. Description of the follow-up period
8. Identification of patients unavailable for statistical analysis (e.g., lost to follow-up)

Each study was assigned a total score ranging from 0 to 8, with higher scores reflecting better quality. Studies scoring greater than 5 were considered to be of adequate overall quality.

### 2.4 Statistical analysis

The current meta-analysis was conducted using RStudio (Posit Software, PBC, Boston, MA, USA; available at https://posit.co/) and the “meta” package (16).

Primary (OS) and secondary (DSS, DFS, LC, LRC, LFS, LEDFS) outcomes were extracted and analyzed as a proportion. For studies in which LSCCs with anterior and posterior extension were analyzed separately (17–19), the outcomes used for the subsequent meta-analysis were calculated as the weighted average of the results for the two extensions. The studies were pooled with both fixed and random effect models, and the weight of each study was calculated with the inverse variance method. Heterogeneity between studies was assessed with the Cochran Q-statistic (*p <*0.05) and I^2^ tests (>50%).

## 3 Results

### 3.1 Literature search results

**Figure 1:**
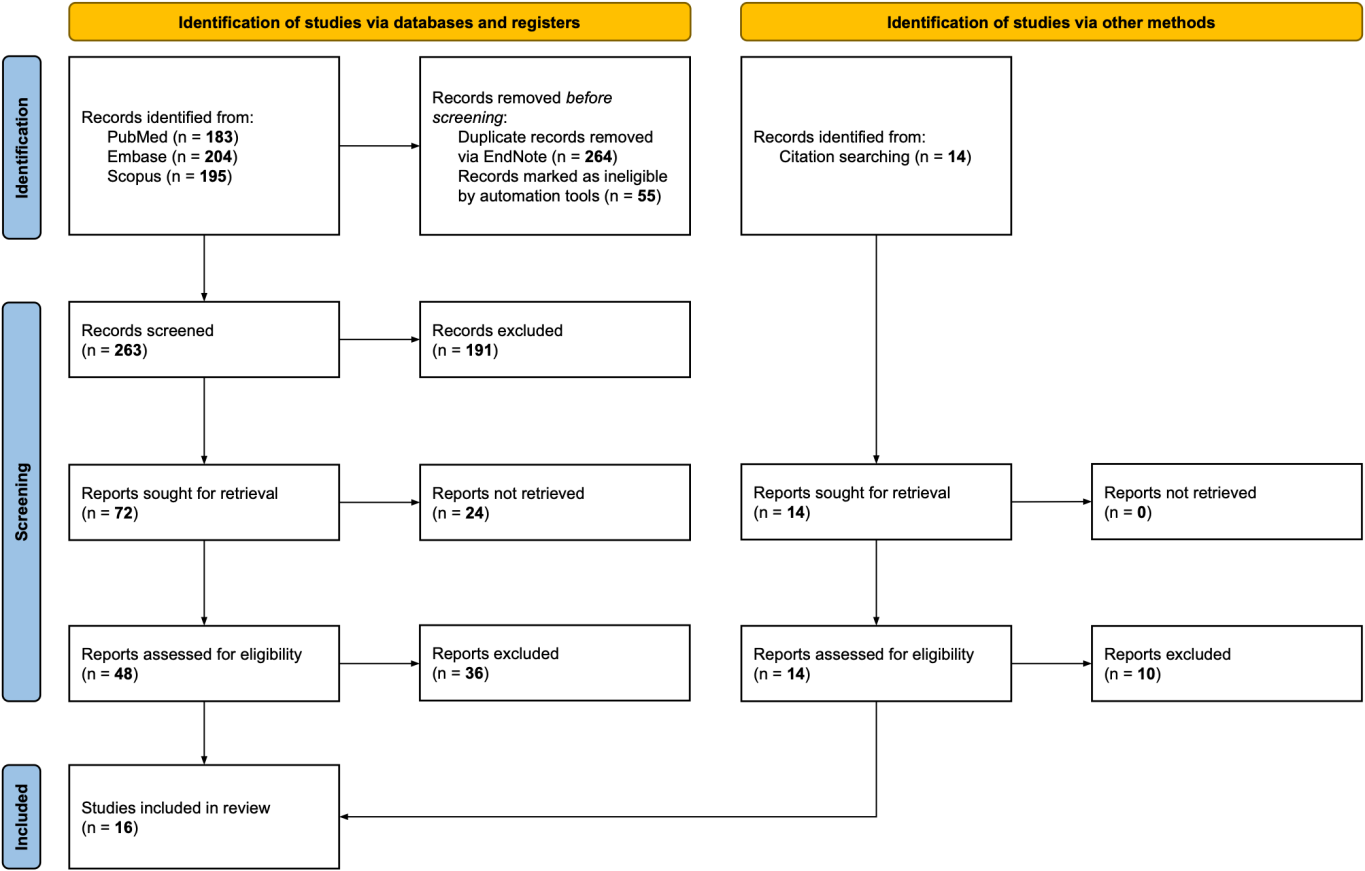
PRISMA 2020 flow diagram.

A flow chart detailing the entire screening process is presented in **Figure 1**. The initial search identified a total of 582 potentially relevant publications. After removing duplicates and excluding records deemed ineligible by automation tools, 263 publications remained. These articles were screened by title and abstract, resulting in the exclusion of 191 documents. Of the remaining records, 24 reports could not be retrieved, and 48 papers underwent full-text evaluation. Citation searches identified 14 additional articles, of which four met the inclusion criteria. The final review included sixteen studies.

### 3.2 Study characteristics

The studies included in this analysis are summarized in **Table 1**.

**Table 1:** Selected studies characteristics.

First Author	Year	Country	Study design	LoE	Study period	Endpoints
Ling (24)	2024	China	retrospective analysis	III	2013–2021	OS, DSS, DFS
Succo (20)	2023	Italy	multi-institutional case series	IV	1995–2019	OS, DSS, DFS, LFS, LEDFS
Zhou (25)	2022	China	retrospective review	III	2005–2010	OS, DSS, DFS
De Vincentiis (17)	2022	Italy	retrospective study	III	2005–2018	OS, DSS, DFS, LFS
Mattioli (21)	2021	Italy	multicentric retrospective cohort study	III	2011–2019	OS, DSS, DFS
Gong (26)	2019	China	retrospective analysis	III	2006–2010	OS, DSS, DFS
Del Bon (18)	2019	Italy	multicentric retrospective cohort study	III	2005–2017	OS, DSS, DFS, LFS
Xia (27)	2018	China	retrospective cohort study	III	1995–2011	OS, DSS, DFS
Succo (19)	2018	Italy	multicentric retrospective study	III	2000–2012	OS, DSS, DFS, LRC, LFS
Zhang (28)	2018	China	retrospective analysis	III	2000–2011	OS
Sperry (31)	2013	United States	retrospective case series	IV	1992–2010	OS, DSS, LC, LRC, LFS
Mercante (22)	2013	Italy	case series	IV	2003–2012	OS, DFS, LRC
Topaloğlu (32)	2012	Turkey	retrospective cohort study	III	2001–2009	OS, DSS
Sánchez-Cuadrado (29)	2011	Spain	case series	IV	1998–2008	OS, DSS, LC
Sevilla (30)	2008	Spain	retrospective study	III	1978–2002	OS
Laudadio (23)	2006	Italy	retrospective analysis	III	1987–1998	OS, DFS

*DFS*, disease-free survival; *DSS*, disease-specific survival; *LC*, local control; *LEDFS*, laryngo-esophageal dysfunction-free survival; *LFS*, laryngectomy-free survival; *LoE*, level of evidence; *LRC*, locoregional control; *OS*, overall survival.

The studies were published between 2006 and 2023. The majority were conducted in Italy (7 studies) (17–23) and China (5 studies) (24–28), with additional studies from Spain (2 studies) (29, 30), the United States (1 study) (31), and Turkey (1 study) (32). Most were retrospective in design (12 of 16) (17–19, 21, 23–28, 30, 32), corresponding to a LoE III, while the remaining 4 were case series (20, 22, 29, 31), representing a LoE IV.

A total of 1473 patients were included. The sample sizes ranged from 17 to 390 patients, and publications spanned 17 years (2006–2023). Patient recruitment occurred between 1988 and 2019. Patients ranged in age from 16 to 90 years. Nodal staging ranged from N0 to N3b. The follow-up period ranged from 0 to 198 months. A targeted search strategy was implemented to ensure that no cases were duplicated across different databases. **Tables 2** and **3** summarize detailed patient and tumor characteristics from the eligible studies.

**Table 2:** Characteristics of patients and tumors in the included studies. .

First Author	Year	No. of patients	T status	N status	R status	Type of laryngectomy	Age (year) (mean)	Age (year) (range)	M:F ratio	Neck dissection	Adjuvant therapy	Follow-up (months) (mean)	Follow-up (months) (range)
Ling (24)	2024	55	pT3	N/A	N/A	OPHL II 53, PVL 2	61.44*	44–80*	(100% M)	N/A	none 30, yes 25	53.51*	7–133*
Succo (20)	2023	134	pT4a	pN0 109, pN1 10, pN2 9, pN3b 6	N/A	OPHL I 2, OPHL IIa 60, OPHL IIb 40, OPHL IIIa 26, OPHL IIIb 6	61.5	41–90	9.31:1	none 4, unilateral 78, bilateral 52	RT 24, CRT 10, CT 1	N/A	N/A
Zhou (25)	2022	108	cT3 105, cT4a 3	cN0	N/A	PVL 64, OPHL IIa 42, OPHL IIb 2	60.8*	30–85*	29.75:1*	N/A	N/A	95.3*	6.6–139.4*
De Vincentiis (17)	2022	116	pT3	N/A	N/A	OPHL II	62.54*	33–79*	14:1*	N/A	N/A	N/A	N/A
De Vincentiis (17)	2022	33	pT4a	N/A	N/A	OPHL II	62.54*	33–79*	14:1*	N/A	N/A	N/A	N/A
Mattioli (21)	2021	28	pT3	pN0	R0	OPHL (I, II, III)	67.1*	46–84*	2.5:1	N/A	RT 4	N/A	N/A
Gong (26)	2019	42	cT3	cN0	N/A	OPHL IIa	57.9*	35–82*	53.67:1*	N/A	N/A	85	12–140
Del Bon (18)	2019	85	pT3 67, pT4a 18	pN1 6, pN2a 5, pN2b 3, pN2c 2, pN3b 2	R0 77, R1 8	OPHL IIa 20, OPHL IIb 42, OPHL IIIa 13, OPHL IIIb 10	60.8	42–78	13:1	ND VI (all), ND II-IV (NS)	RT 13, CRT 5	55.1	6–148
Xia (27)	2018	106	cT3	N/A	N/A	OPHL II	58.7	34–70	9.6:1	N/A	none 57, RT 49	72.6	6–184*
Succo (19)	2018	390	pT3	N0 336, N1 23, N2 31	N/A	OPHL IIa 242, OPHL IIb 111, OPHL IIIa 37	60.0*	16–83*	9.64:1*	N/A	N/A	63.6*	6–196.8*
Succo (19)	2018	89	pT4a	N0 71, N1 8, N2 10	N/A	OPHL IIa 28, OPHL IIb 30, OPHL IIIa 24, OPHL IIIb 7	60.0*	16–83*	9.64:1*	N/A	N/A	63.6*	6–196.8*
Zhang (28)	2018	32	pT4a	pN1 13, pN2 19	N/A	OPHL IIb	54.8	45–62	9.67:1	unilateral 13, bilateral 19	RT 27, CRT 5	78	63–95
Sperry (31)	2013	28	pT3	cN0 20, cN1 3, cN2b 3, cN2c 2	N/A	OPHL II	60*	33–78*	9.8:1*	none 2, unilateral 15, bilateral 11	N/A	51*	0–198*
Mercante (22)	2013	32	cT3	cN0 27, cN1 3, cN2b 2	R0 (all)	OPHL IIa 14, OPHL IIb 18	62	24–80	15:1	unilateral SND 17, bilateral SND 9, SND + mRND 4, unilateral mRND 1	RT 2, CRT 3	47.3	6–116
Topaloğlu (32)	2012	27	pT3	pN0 16, pN1 2, pN2a 3, pN2b 1, pN2c 4, pN3 1	N/A	OPHL IIb	55.48	44–75	26:1	bilateral mRND (pN1-pN2) 26, left mRND + right RND (pN3) 1	RT 11	47.3	5–83
Saánchez-Cuadrado (29)	2011	17	T3	N0 14, N1 1, N2 1, N3 1	N/A	OPHL II	56*	38–71*	(100% M)	N/A	N/A	43*	12–120*
Sevilla (30)	2008	80	pT3 49, pT4 31	N/A	N/A	OPHL I	58*	33–79*	16.8:1*	N/A	N/A	45.6*	N/A
Laudadio (23)	2006	71	pT3 58, pT4 13	pN0 61, pN1 7, pN2 3	N/A	OPHL IIa 58, OPHL IIb 13	62.2*	44–78*	33.33:1*	N/A	N/A	62*	3–156*

*CRT*, chemoradiotherapy; *CT*, chemotherapy; *mRND*, modified radical neck dissection; *N/A*, not available; *ND*, neck dissection; *NS*, not specified; *OPHL*, open partial horizontal laryngectomy; *PVL*, partial vertical laryngectomy; *RND*, radical neck dissection; *RT*, radiotherapy; *SND*, selective neck dissection.

Some studies were further stratified when data were available for specific T stages (T3 vs. T4a).

Data marked with an asterisk (*) indicate instances where subpopulation-specific references could not be found; therefore, values for the entire study cohort are presented.

**Table 3:** Five-year survival and functional outcomes in T3 and T4a LSCC patients undergoing partial laryngectomy in the included studies.

First Author	Year	T status	OS (%)	DSS (%)	DFS (%)	LC (%)	LRC (%)	LFS (%)	LEDFS (%)
Ling (24)	2024	pT3	91.6	95.8	79.2	N/A	N/A	N/A	N/A
Succo (20)	2023	pT4a	82.1	89.8	75.7	N/A	N/A	89.7	78.3
Zhou (25)	2022	cT3, cT4a	78.5	79.3	65.4	N/A	N/A	N/A	N/A
De Vincentiis (17)	2022	pT3	79.3	85.3	81	N/A	N/A	82.76	N/A
De Vincentiis (17)	2022	pT4a	70.9	77.4	77	N/A	N/A	66.67	N/A
Mattioli (21)	2021	pT3	92.9	100	89.3	N/A	N/A	N/A	N/A
Gong (26)	2019	cT3	77.8	77.8	63.3	N/A	N/A	N/A	N/A
Del Bon (18)	2019	pT3, pT4a	74.1	79.1	59.4	N/A	N/A	59.9	N/A
Xia (27)	2018	cT3	65.8	73.6	72.1	N/A	N/A	N/A	N/A
Succo (19)	2018	pT3	90.1	94.5	87.4	N/A	88.8	86.8	N/A
Succo (19)	2018	pT4a	81.9	91.3	71.2	N/A	75.5	72.9	N/A
Zhang (28)	2018	pT4a	62.5	N/A	N/A	N/A	N/A	N/A	N/A
Sperry (31)	2013	pT3	78	81	N/A	96	82	91	N/A
Mercante (22)	2013	cT3	87.3	N/A	78.2	N/A	96.2	N/A	N/A
Topaloğlu (32)	2012	pT3	87	91.4	N/A	N/A	N/A	N/A	N/A
Sánchez-Cuadrado (29)	2011	T3	52	64	N/A	67	N/A	N/A	N/A
Sevilla (30)	2008	pT3, pT4	65	N/A	N/A	N/A	N/A	N/A	N/A
Laudadio (23)	2006	pT3, pT4	78.9	N/A	73.2	N/A	N/A	N/A	N/A

*DFS*, disease-free survival; *DSS*, disease-specific survival; *LC*, local control; *LEDFS*, laryngo-esophageal dysfunction-free survival; *LFS*, laryngectomy-free survival; *LRC*, locoregional control; *N/A*, not available; *OS*, overall survival.

Some studies were further stratified when data were available for specific T stages (T3 vs. T4a).

### 3.3 Quality assessment

The quality assessment scores of the studies ranged from 5 to 7 based on the REMARK criteria, summarized in **Table 4**. The median score was 7, and the mean was 6.5. Of the 16 included studies, 15 received scores greater than 5, indicating that most were of adequate quality.

**Table 4:** Quality assessment scores of the studies included.

First Author	Year	Inclusion and exclusion criteria	Prospective/retrospective	Patient characteristics	Tumor characteristics	Margins status definition	Endpoint	Follow-up period	Patients unavailable for statistical analysis	Quality scale
Ling (24)	2024	1	1	1	1	1	1	1	0	7
Succo (20)	2023	1	1	1	1	1	1	0	1	7
Zhou (25)	2022	1	1	1	1	1	1	1	0	7
De Vincentiis (17)	2022	1	1	1	1	1	1	0	1	7
Mattioli (21)	2021	1	1	1	1	1	1	0	0	6
Gong (26)	2019	0	1	1	1	1	1	1	0	6
Del Bon (18)	2019	1	1	1	1	1	1	1	0	7
Xia (27)	2018	1	1	1	1	0	1	1	0	6
Succo (19)	2018	1	1	1	1	1	1	1	0	7
Zhang (28)	2018	1	1	1	1	0	1	1	0	6
Sperry (31)	2013	0	1	1	1	1	1	1	1	7
Mercante (22)	2013	0	1	1	1	1	1	1	0	6
Topaloğlu (32)	2012	0	1	1	1	0	1	1	0	5
Sánchez-Cuadrado (29)	2011	0	1	1	1	0	1	1	1	6
Sevilla (30)	2008	1	1	1	1	1	1	1	0	7
Laudadio (23)	2006	1	1	1	1	0	1	1	1	7

### 3.4 Oncological outcomes and meta-analysis

**Figure 2:**
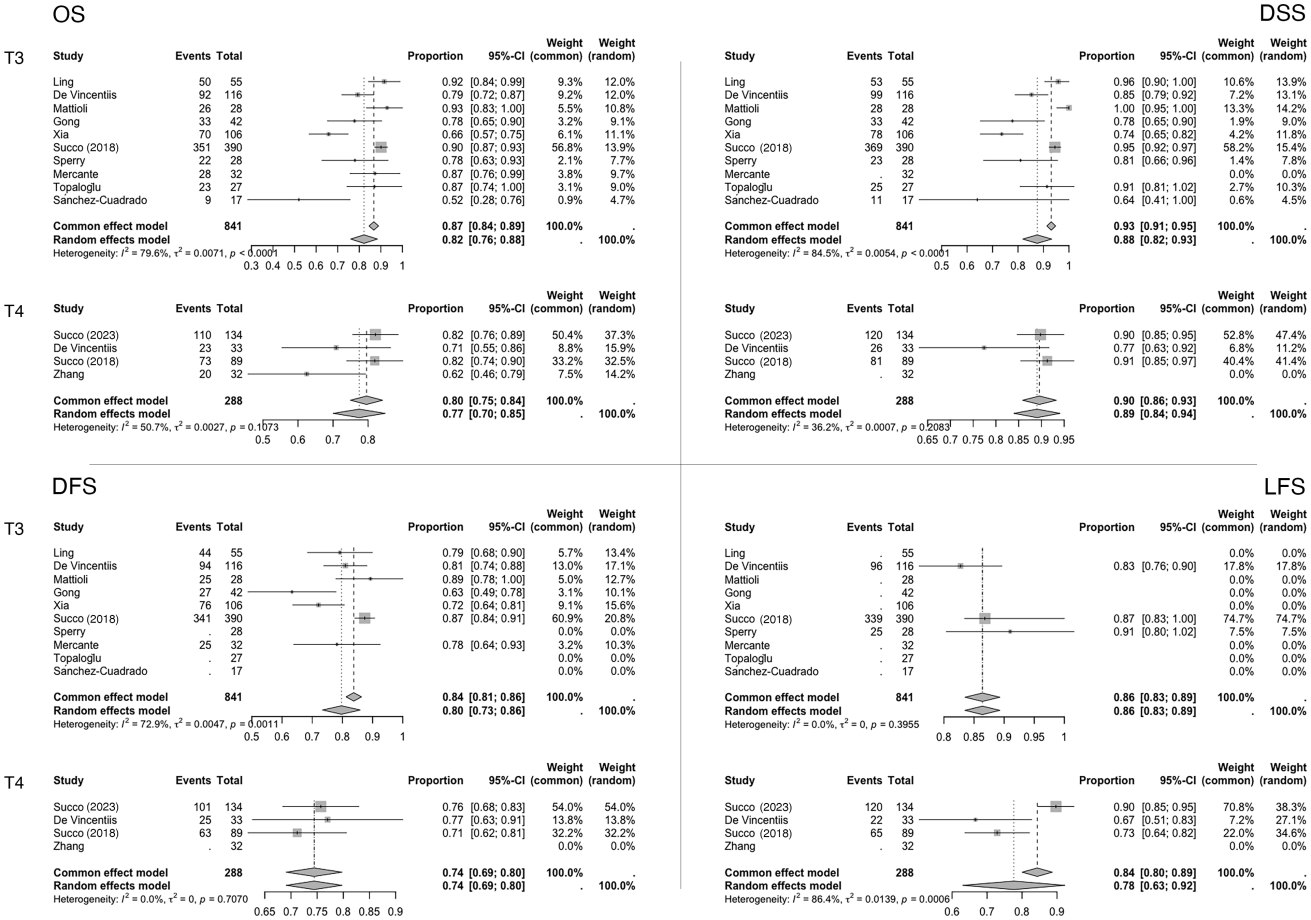
Meta-analysis forest plots. Data are reported as proportions (events over total) ± 95% CI. The weight of the various articles in the fixed and random effects model is also highlighted.

Variability in the reported outcomes across the included studies precluded statistical analyses and meta-analyses for several secondary endpoints. Specifically, LC, LRC, and LEDFS data were insufficiently reported in the T3 or T4a case series. Consequently, the meta-analysis focused on OS, DSS, DFS, and LFS (**Figure 2**). Although fixed-effect estimates are presented, the results should primarily be interpreted under the random-effects model due to the limited number and/or marked heterogeneity of the included studies. The near-zero value of the τ^2^ estimator in the meta-analysis suggests negligible variability in effect sizes among these studies under the random-effects model.

For the primary endpoint (five-year OS), the pooled proportion in the T3 case series was 0.82 (95% CI, 0.76–0.88; I²=79.6%; N=841), while in the T4a case series, it was 0.77 (95% CI, 0.70–0.85; I²=50.7%; N=288).

At five years, the pooled DSS proportions were 0.88 (95% CI, 0.82–0.93; I²=84.5%; N=809) for T3 patients and 0.89 (95% CI, 0.84–0.94; I²=35.2%; N=256) for T4a patients. Similarly, the pooled DFS proportions were 0.80 (95% CI, 0.73–0.86; I²=72.9%; N=769) for T3 patients and 0.74 (95% CI, 0.69–0.80; I²=0.0%; N=256) for T4a patients.

Finally, for the functional outcome LFS at five years, the pooled proportions were 0.86 (95% CI, 0.83–0.89; I²=0.0%; N=534) in T3 case series and 0.78 (95% CI, 0.63–0.92; I²=86.4%) in T4a case series.

Not all studies reported the gastrostomy rate at a follow-up period after the OPHL procedure. When reported, the presence of permanent gastrostomy was observed in 12 out of 919 cases (1.31%).

Regarding the total laryngectomy rate, most studies reported only the LFS without clarifying whether total laryngectomy was performed for oncologic reasons or as a completion laryngectomy due to persistent dysfunctional sequelae. In the studies where this information was explicitly provided, 27 out of 276 patients undergoing OPHL (9.78%) eventually required a total laryngectomy, all for oncologic indications.

## 4 Discussion

When multiple therapeutic options for a specific condition yield comparable survival outcomes, assessing the impact of each option on post-treatment functionality becomes essential. In the management of LSCC, there has been a resurgence of interest in partial laryngeal surgery, particularly OPHL, as a viable unimodal therapeutic option, serving both as an alternative to total laryngectomy and as an organ-preserving approach compared to radiotherapy or chemoradiotherapy (33). This meta-analysis shows that OPHL has significantly enhanced the conservative treatment of intermediate (T3) and locally advanced (T4a) LSCC, providing stable and reliable results. OPHL presents a safe option for treatment-naïve patients with T3 LSCC, achieving a five-year pooled proportion of OS, DSS, DFS, and LFS at 0.82, 0.88, 0.80, and 0.86, respectively. Similar results were seen in treatment-naïve T4a patients, with five-year OS, DSS, DFS, and LFS rates averaging 0.77, 0.89, 0.74, and 0.78, respectively. As highlighted by Succo et al. (9), since many pT4a tumors result from upstaging of cases initially staged as cT3, OPHL provides comprehensive coverage, effectively managing cases with a high risk of upstaging in the pathological report. Aggregated oncologic data across studies indicate that OPHL may obviate the need for total laryngectomy, preserving essential laryngeal functions.

Thus, DSS and LRS outcomes reflect an increasingly refined patient selection process. Over time, this has helped identify those who benefit most from surgical organ preservation, reducing the role of upfront total laryngectomy and positioning surgical organ preservation as a strong alternative to chemoradiotherapy-based (CRT) organ preservation protocols.

Undoubtedly, DSS outcomes with OPHL are impressive, surpassing those of comparable cohorts treated with total laryngectomy or CRT-based organ preservation. Given that lower local control rates or DSS in total laryngectomy cohorts compared to OPHL cohorts seem unlikely, this discrepancy likely stems from differences in patient selection. Patients chosen for surgical organ preservation differ significantly in several factors, including age (generally younger in surgical organ preservation), fitness level (Karnofsky performance status [KPS] ≥90), and disease stage (typically cN0 or cN1) or more favorable cases (T3 or T4a without extension into the posterior paraglottic space or invasion of the cricoarytenoid unit, a factor associated with worse prognosis and DSS outcomes similar to those of total laryngectomy). It is evident that surgical organ preservation, even more than CRT-based organ preservation, is directed toward highly selected cases. However, due to its excellent oncologic outcomes, it significantly reduces the total laryngectomy rate within this subset of patients.

Among the reviewed studies, when reported, OPHL type II (supracricoid laryngectomy) was the most common form of partial laryngectomy (1174 of 1445 patients), followed by OPHL type III (supratracheal laryngectomy, 123 patients) and type I (supraglottic laryngectomy, 82 patients). Regarding patient selection based on N status, in studies where it is determinable, the majority were N0 (832 of 1083; 76.8%), while 23.2% were N+. The sample was relatively homogeneous in both treatment type (OPHL) and clinical staging, predominantly involving intermediate stages (T3) and only rarely locally advanced stages (T4a), with a low incidence of lateral cervical lymph node metastases.

Despite the excellent OS, LC, and LFS outcomes reported in the literature, functional preservation protocols involving OPHL have not been widely adopted due to a lack of standardized patient selection criteria and variability in functional outcomes. According to NCCN guidelines (34), OPHL is recommended for highly selected patients as an alternative to non-surgical treatment for glottic and supraglottic LSCCs at stages T1N0, T2N0, and selected T3N0 cases. Few studies have defined specific selection criteria for OPHL for patient and tumor characteristics. The main contraindications to OPHL for T3 tumors include invasion of the posterior paraglottic space with cricoarytenoid joint and cricoid involvement. A subglottic extension of less than 10 mm at the vocal cord midline and a nodal stage above cN1 are considered relative contraindications and should be evaluated in a multidisciplinary setting (35).

Succo et al. (19) demonstrated the fundamental role of anatomical and functional compartmentalization of the larynx into anterior and posterior regions based on the transgression of a “magic” frontal plane crossing the arytenoid vocal process and the ipsilateral thyroid lamina. This anatomical (endoscopic and radiological) observation, combined with the fixation status of the arytenoid cartilage, appears to have a predictive value exceeding that of the traditional TNM classification, particularly for the highly heterogeneous T3 stage, with varied anatomical-functional characteristics and diffusion pathways. In 479 cases, the authors found that posterior T3 tumors with arytenoid fixation exhibit oncologic outcomes similar to T4a tumors. Posterior T3 tumors, which spread into the posterior paraglottic space causing arytenoid fixation, show worse oncologic outcomes with OPHL compared to anterior tumors (OS *p <*0.001, DSS *p <*0.05, and DFS *p <*0.001). Other studies have corroborated that anterior T3 tumors have superior oncologic outcomes compared to posterior tumors. To a lesser extent, this is also seen in pT4a tumors with minimal extralaryngeal extension.

Recent multicenter analyses have supported the conservative management of pT4a tumors, often considered anecdotal and limited to cases with minimal extralaryngeal extension. For pT4a tumors with minimal extralaryngeal volume treated with OPHL type II and type III, oncologic outcomes are consistent with those seen in pT3 cases (17).

The NCCN guidelines (31) recommend adjuvant radiotherapy for patients with N2 or N3 lymph node metastases. While radiotherapy techniques have advanced to reduce tissue damage, postoperative radiotherapy may still impair functional outcomes (36), potentially leading to dysphagia, tissue necrosis, laryngeal edema, xerostomia, fibrosis, and reduced quality of life. Consequently, unimodal treatment is preferable. OPHL is generally not recommended for patients with lymph node metastases above cN1 due to the high risk of locoregional failure and the probable need for adjuvant therapy. The subglottic extension also requires careful assessment to ensure adequate margins and avoid additional treatments.

Most patients included (639 patients) were T3N0. This review did not exclusively analyze pN0 patients; **Table 2** shows that some authors also included cases with lymph node metastases, though these were rare and primarily in supraglottic tumors.

A total of 499 patients (33.9%) received adjuvant radiotherapy. A limitation of this study is that the precise indications for adjuvant radiotherapy in 179 of the 499 patients could not be determined. The total of 499 patients refers only to those for whom information on the use or non-use of postoperative radiotherapy was available, as not all included studies reported this data comprehensively. Furthermore, among the studies that did report on adjuvant treatments, only the total number of patients who underwent radiotherapy was provided, without specifying their classification as T3 or T4a or detailing the specific indications for the treatment. It could be argued that the need for adjuvant therapies after OPHL suggests less-than-ideal case selection for surgical organ and function preservation, as multimodal therapy often compromises functional outcomes. Ideally, rigorous patient selection and surgical intervention with sufficient margins would eliminate the need for adjuvant radiotherapy.

Furthermore, NCCN guidelines distinguish T3 patients who are candidates for OPHL from those who are not and instead require total laryngectomy (34). T3 patients undergoing OPHL may have characteristics differing from those in the total laryngectomy group—even when classified as pT3N0—potentially reflecting variations in selection criteria across centers. Randomized studies comparing OPHL and total laryngectomy for pT3N0 patients do not exist in the literature, and conducting such studies would be nearly impossible.

The biological criteria for identifying optimal OPHL candidates include good psychophysical and family support (including caregivers), good overall health as indicated by the ability to climb two flights of stairs (4 metabolic equivalents [METs]), and the absence of significant comorbidities (37). A rigorous rehabilitation protocol is essential for functional recovery of the neolarynx, with caregiver support crucial for nutritional assistance and reintegration into daily life. Additionally, general health must be thoroughly assessed, as neurological, pulmonary, and cardiovascular comorbidities may contraindicate OPHL due to subclinical aspiration and pneumonia risk.

Prognostication of functional outcomes post-OPHL is critical for surgical planning, necessitating a thorough evaluation of preoperative factors associated with both patient and disease to enable personalized treatment strategies. Functional recovery, particularly regarding voice, breathing, and swallowing, is generally satisfactory but varies across patients, with voice quality being difficult to predict. These procedures help preserve essential laryngeal functions, eliminating the need for a permanent tracheostomy. Laryngeal function preservation rates five years post-OPHL range between 91.2% and 98.5%, depending on primary disease extension (38, 39). Identifying prognostic factors for complex functional recoveries is essential for optimizing preoperative patient selection. Voice quality is best preserved with OPHL type I due to the conservation of the vocal cord. In contrast, OPHL type II and type III result in significant voice degradation, although they still permit acceptable oral communication through substitution voice techniques (40). Swallowing function following OPHL type II has been extensively investigated in the literature (41–43). In the immediate postoperative phase, nearly all patients experience dysphagia, with the incidence approaching 100%. However, spontaneous recovery generally occurs within 3 to 6 months, allowing most individuals to resume an unrestricted oral diet. Nonetheless, long-term issues—such as chronic aspiration, particularly with liquids, and post-swallow residue, especially with solids—are commonly reported in the literature, even though these complications do not result in a high rate of permanent gastrostomy.

Studies comprehensively assessed laryngeal function, with promising data for LFS and LEDFS (a composite functional endpoint). Succo et al. report LFS at 86.8% and 72.9% for pT3 and pT4a, respectively, and an LEDFS of 78.3% in a multi-institutional series of pT4a cases (19).

Recently, attention has shifted from exclusively oncologic outcomes to including functional outcomes in partial laryngectomies, focusing on post-OPHL quality of life. Functional results do not always meet initial expectations; however, several rehabilitative and surgical interventions can significantly enhance laryngeal function—namely, respiratory, swallowing, and phonatory capabilities (44). Phonosurgical injection techniques have shown promise in improving voice quality and swallowing, and transoral laser microsurgery has effectively managed laryngeal stenosis post-OPHL (45–47). The proprioceptive elastic method (PROEL) has proven beneficial for substitute voice rehabilitation (48). Integrating these tailored approaches, based on individual patient needs, is vital for optimizing functional outcomes.

Although OPHLs are applied only to carefully selected cases, they often provide superior functional preservation compared to both total laryngectomy and organ preservation treatments. Patients undergoing total laryngectomy generally achieve satisfactory swallowing restoration. Nonetheless, despite advances in surgical techniques and rehabilitation—including tracheoesophageal puncture for voice restoration—total laryngectomy invariably results in a permanent tracheostoma, adversely affecting respiratory function and overall quality of life (49). Organ preservation treatments, while sparing the laryngeal structure, often lead to long-term functional impairments due to radiation-induced fibrosis and neuromuscular dysfunction. Swallowing difficulties, chronic aspiration, and dietary restrictions are common among these patients, with some studies reporting up to 40% of cases requiring salvage surgery due to local failure. Additionally, while organ preservation may initially seem advantageous, post-treatment dysphagia and speech impairment can significantly affect long-term functionality (50). Conversely, the optimal unimodal surgical approach provided by OPHLs circumvents the late toxic effects associated with radiotherapy while preserving a functional neoglottis that supports both deglutition and phonation. Long-term studies indicate that, although patients undergoing OPHL initially experience dysphagia, these individuals typically recover a stable swallowing function that remains relatively well preserved with aging (51).

A significant limitation of this review is that all included articles are retrospective and subject to inherent biases. Although heterogeneity in meta-analyses remains a limitation, the inclusion of 1473 patients with T3 and T4a LSCCs treated with partial laryngectomy over the past 20 years reinforces these findings. Given the absence of heterogeneity among effect sizes coupled with considerable overall variation, it is plausible that the observed heterogeneity among studies cannot be attributed solely to chance but may reflect variations in patient selection and treatment application.

## 5 Conclusions

Partial laryngectomies represent a safe and effective surgical option for LSCC, particularly for treatment‐naïve T3N0 patients, yielding excellent oncologic and functional outcomes when patients are carefully selected. Morbidity and mortality rates are within acceptable ranges, and modern approaches to tumor selection and adaptable resection techniques facilitate the management of cases that are upstaged from cT3 to pT4a, provided that extralaryngeal extension is minimal. The primary aim remains to use a single-modality approach to preserve both organ and laryngeal function, avoiding the need for total laryngectomy at these stages. Although standard functional assessments demonstrate favorable outcomes, more refined measures are required to comprehensively compare OPHL with alternative treatment modalities.

OPHL remains a highly specialized and infrequently performed procedure worldwide. Although it may not capture the routine interest of the general ENT surgeon, the robust oncological and functional outcomes documented in numerous series—even in T3 and T4a cases—affirm this organ-preserving strategy as one of the most effective alternatives to total laryngectomy for carefully selected cases.
